# Development and validation of an interpretable prediction model using spatial patterns of tumor-infiltrating lymphocytes in H&E-stained whole-slide images for immune subtyping of lung adenocarcinoma

**DOI:** 10.3389/fimmu.2026.1773927

**Published:** 2026-05-08

**Authors:** Xia Li, Hai-Zhen Qin, Jing-Yu Wei, Kang-Lai Wei, Zhao-Quan Huang

**Affiliations:** 1 Department of Pathology, The First Affiliated Hospital of Guangxi Medical University, Nanning, Guangxi, China; 2 Department of Pathology, The Second Affiliated Hospital of Guangxi Medical University, Nanning, Guangxi, China

**Keywords:** computational pathology, immune subtyping, interpretable model, lung adenocarcinoma, tumor-infiltrating lymphocytes

## Abstract

**Objective:**

To develop an interpretable prediction model for lung adenocarcinoma immune subtyping by quantifying spatial distribution patterns of tumor-infiltrating lymphocytes in H&E whole-slide images, providing a computational tool for tumor immune microenvironment evaluation.

**Methods:**

Immune subtyping was performed on the TCGA lung adenocarcinoma cohort using ssGSEA to quantify immune gene set activity, followed by hierarchical clustering and t-SNE visualization to stratify patients into high- and low-immunity subgroups. Immune cell infiltration was assessed using CIBERSORT, while tumor mutation burden and somatic mutation profiles were analyzed with maftools. Differential expression and functional enrichment analyses were conducted using GO and KEGG databases. In pathological image analysis, an automated annotation model optimized with study-specific data was employed to process whole-slide images. Immune subtype prediction criteria were established by quantifying spatial distribution features of tumor-infiltrating lymphocytes. The model’s predictive performance was validated in both internal and external cohorts.

**Results:**

Transcriptomic analysis stratified 503 LUAD patients into high- and low-immunity subgroups. The high-immunity group exhibited elevated infiltration of CD8^+^T cells and M1 macrophages, higher tumor mutation burden, and enriched T cell activation pathways. The low-immunity group showed predominant resting immune cells. The automated annotation model, achieved a 95.09% Dice score for tissue contour segmentation, 91.53% for tumor parenchyma segmentation, and a 79.51% F1-score with an mAP@0.5 of 82.13% for TIL identification. Quantitative TIL spatial distribution analysis with a 0.2 high-attention threshold enabled development of an immune subtyping model using a 0.05 classification cutoff, which achieved an AUC of 0.839 for immune subtype classification in the internal validation cohort. In the external validation cohort, the model achieved an AUC of 0.927, and immunohistochemical analysis confirmed significantly higher densities of CD3^+^, CD8^+^, CD20^+^, and CD68^+^ cells in predicted high-immunity samples.

**Conclusion:**

This study establishes an interpretable immune subtype prediction model for LUAD based on TIL spatial distribution in H&E-stained sections. Through a modular design that integrates deep learning-based annotation with statistical classification, the model links morphological phenotypes to molecular immune subtypes while maintaining transparency and verifiability throughout the analytical workflow. This cost-effective and scalable tool offers potential value for assessing tumor immune status and guiding immunotherapy decision-making.

## 1 Introduction

Assessment of tumor immune microenvironment(TIME) heterogeneity is closely associated with immunotherapy response and patient prognosis (1, 2). This evaluation is particularly critical in lung adenocarcinoma (LUAD), the most common pathological subtype of non-small cell lung cancer (3). LUAD exhibits persistently high global incidence and mortality rates, with advanced-stage patients facing poor prognosis, making it a leading cause of cancer-related deaths (4, 5). Although immune checkpoint inhibitors have been widely adopted in LUAD treatment (6), their efficacy depends heavily on the status of the TIME. The overall response rate to immunotherapy in LUAD patients remains below 20% (7), highlighting the urgent need to dissect TIME heterogeneity and establish a clinically applicable classification system. Such a system is crucial for precisely identifying potential immunotherapy beneficiaries, formulating individualized treatment strategies, and improving patient survival outcomes.

Within the complex immune microenvironment of LUAD, tumor-infiltrating lymphocytes (TILs) are predominantly located in stromal regions. As core effector cells of TIME, TILs play a key role in immune status regulation. Abundant lymphocyte infiltration often associates with an activated immune phenotype, correlating with better immunotherapy response and improved survival outcomes (8, 9). Conversely, absence of lymphocyte infiltration typically indicates an immunosuppressed or tolerant state, often accompanied by poor immunotherapy response and unfavorable clinical outcomes (10). This evidence demonstrates that the extent and spatial distribution of lymphocyte infiltration can drive functionally distinct immune subtypes, making TIL evaluation one of the most direct and biologically meaningful measures of TIME heterogeneity.

Transcriptomic analysis provides a reliable method for distinguishing immune subtypes, enabling comprehensive assessment of immune-related gene expression, cellular composition, and functional states while overcoming subjective interpretation biases at the molecular level (11). However, the high cost of transcriptome sequencing and stringent sample requirements limit its routine clinical application. Additionally, transcriptomic data reflect bulk-tissue molecular expression profiles and cannot resolve the spatial distribution of immune cells *in situ*. Growing evidence emphasizes the critical role of immune cell spatial organization in immune regulation and treatment response prediction (12, 13). In contrast, hematoxylin and eosin (H&E) staining represents the most routine and cost-effective diagnostic method in oncology, enabling assessment of both immune cell infiltration levels and spatial distribution within the tumor microenvironment. However, current evaluation primarily relies on pathologists’ visual semi-quantitative assessment, which suffers from subjectivity, poor inter-observer consistency, and limited precision in quantifying complex spatial patterns.

Recent integration of digital pathology and artificial intelligence (AI) has provided new methodologies for cross-modal data research in oncology. Deep convolutional neural networks enable automated feature extraction from whole-slide images (WSIs). This automated feature extraction capability grants them significant advantages in tasks such as object detection and tissue segmentation, allowing them to autonomously learn and recognize complex morphological patterns from large-scale pathological images without the need for manually designed features. Furthermore, by capturing the complex nonlinear relationships between morphological features and molecular expression, deep learning methods provide powerful tools for exploring associations between morphological phenotypes and molecular expression. Several studies have leveraged this advantage to directly predict molecular features from WSIs using AI algorithms (14, 15), demonstrating connections between morphological and molecular phenotypes. Other studies have employed deep learning models to analyze relationships between TILs and patient survival (16, 17). However, most existing models adopt end-to-end architectures lacking biological interpretability (18), and research correlating H&E image features with transcriptome-based immune subtyping remains limited.

This study presents a modular computational pathology framework that establishes a mapping between histopathological features and transcriptomic immune subtypes in LUAD. The framework adopts a modular design that separates automated annotation from spatial analysis, in which the annotation module leverages a deep learning tool from our previous work to generate large-scale annotated data, while the quantification module introduced in this study performs statistics-driven analysis of TILs spatial patterns and maps them to immune subtypes. This approach enables accurate prediction of transcriptome-based immune subtypes directly from H&E images. The modular architecture ensures transparency throughout the analytical workflow, with intermediate outputs directly verifiable by pathologists. By confining deep learning to the annotation task and employing interpretable statistical rules for classification, this framework effectively addresses the “black box” limitation of end-to-end models, enhancing clinical trustworthiness with important implications for LUAD patient stratification and immunotherapy decision-making.

## 2 Data and methods

### 2.1 Data sources and preprocessing

#### 2.1.1 Transcriptomic data

RNA sequencing data and corresponding clinical information from 503 LUAD tissue samples and 54 matched normal tissue samples were obtained from The Cancer Genome Atlas (TCGA) Data Portal (https://portal.gdc.cancer.gov/). To ensure data quality and comparability, raw read counts were converted to transcripts per million (TPM) values, and batch effect correction was performed using the “limma”and “sva” R packages.

#### 2.1.2 Whole-slide image data

From the 503 LUAD cases in TCGA, 314 available WSIs were initially identified. Then, two pathologists independently evaluated all WSIs and applied the following exclusion criteria:

1. Abnormal staining (e.g., over- or under-stained);
2. Compromised tissue integrity (e.g., folds, overlaps, or tears);
3. Image artifacts (e.g., handwritten marks, blurring, or insufficient magnification);
4. Insufficient tissue area;
5. Absence of formalin-fixed paraffin-embedded (FFPE) sections (frozen sections only);
6. Non- H&E staining.

Cases with non-LUAD pathological types or >30% missing key clinical data were also excluded. Furthermore, any image region containing less than 60% tissue area was discarded to minimize background interference during model training.

### 2.2 Study cohort definition

Three independent cohorts were established for model development and validation. The training set comprised 80 TCGA-LUAD cases with complete transcriptomic immune subtyping and automated WSI annotations. The internal validation set included 100 rigorously quality-controlled TCGA-LUAD cases (43 high-immunity, 57 low-immunity), independently selected with no overlap to the training set. An external validation cohort was retrospectively established using 60 LUAD patients from the Second Affiliated Hospital of Guangxi Medical University, selected based on these criteria:

Inclusion criteria:

1. Histopathologically confirmed primary pure LUAD (ICD-10: C34.90) diagnosed as “invasive lung adenocarcinoma” without other histological components;
2. Age ≥18 years;
3. Hospitalization between October 1, 2020 and July 31, 2025 with complete baseline clinical data.

Exclusion criteria:

1. Other primary lung cancer types or metastatic adenocarcinoma;
2. Missing ≥30% key clinical data or unavailable pathological materials;
3. History of other malignancies within 5 years;
4. Comorbid autoimmune diseases, immunosuppressive therapy, or prior immunotherapy;
5. Pregnancy, lactation, or participation in interfering clinical trials.

A total of 60 LUAD patients were included as an external validation cohort based on the above criteria. All samples were processed through the automated annotation pipeline constructed in this study, and the immune phenotype prediction model performed subtype predictions based on the output TILs spatial distribution data. This study followed the Declaration of Helsinki and was approved by the hospital ethics committee. As this was a retrospective study, the requirement for written informed consent was waived.

All cases across cohorts had complete baseline clinical data including age, sex, and TNM stage (AJCC 8th edition).

### 2.3 Construction of immune subtypes based on transcriptomic data

A validated set of immune-related genes was obtained from the ImmPort immunology database. Gene set annotation files were processed using the “GSEABase” package, and the single-sample gene set enrichment analysis (ssGSEA) algorithm from the “GSVA” package was applied to calculate immune signature enrichment scores for each sample based on the standardized LUAD gene expression matrix from TCGA.

Immune subtyping was performed using a combined approach of hierarchical clustering and nonlinear dimensionality reduction based on the ssGSEA-derived immune signature score matrix. Hierarchical clustering analysis was first conducted with the “sparcl” package to determine the optimal cluster number. The t-SNE dimensionality reduction algorithm was then implemented using the “rtsne” package to project high-dimensional immune features into two-dimensional space. This analysis categorized all samples into two distinct immune subtypes: high-immunity and low-immunity groups. Visualization was performed using the “ggplot2” package, with high- and low-immunity groups colored red and blue, respectively. To comprehensively display intergroup differences in immune signatures, gene sets were Z-score normalized, and a clustered heatmap was generated using the “pheatmap” package.

### 2.4 Multi-dimensional bioinformatics validation of transcriptome-based immune subtyping

#### 2.4.1 Analysis of immune cell infiltration patterns

The CIBERSORT algorithm was applied to evaluate differences in immune cell composition between high- and low-immunity subgroups. Deconvolution analysis was performed for each sample, and the relative proportions of 22 immune cell types were normalized to sum to 1. Distribution patterns of immune cell subsets across subgroups were visualized using boxplots generated with the “ggpubr” package.

#### 2.4.2 Tumor mutational burden and genomic alteration analysis

Whole-exome sequencing data in MAF format were obtained from the TCGA-LUAD cohort. Tumor mutation burden (TMB) was calculated using the “maftools” R package, defined as the number of non-synonymous mutations per megabase. Differential TMB analysis between immunity subgroups was performed using the “limma” package, with results visualized via “ggplot2” boxplots. Somatic mutation landscapes were characterized by analyzing frequently mutated genes, mutation types, and single nucleotide variant (SNV) patterns separately for each subgroup.

#### 2.4.3 Functional enrichment analysis

Differentially expressed genes between immunity subgroups were identified using the “limma” package (thresholds: |log2FC| ≥ 1.2, FDR < 0.05). Functional enrichment analysis was performed using the “clusterProfiler” package, including Gene Ontology (GO) and KEGG pathway analyses.

### 2.5 Automated annotation pipeline development and training

The automated annotation pipeline used in this study was derived from a separate systematic methodological investigation by our team (19). This pipeline encompasses a complete data annotation pipeline, preprocessing strategies, and core algorithms, originally developed as a standardized tool for digital pathology image annotation. Here, we applied this pipeline to generate large-scale, high-quality morphological annotations for subsequent immune subtyping analysis. The following section provides a brief overview of its workflow and key technical features.

#### 2.5.1 Manual annotation and preprocessing

To construct a high-quality training dataset for this automated annotation model, a subset of WSIs was randomly selected from eligible TCGA cases. Two pathologists with over three years of experience in thoracic oncology pathology independently performed annotations using QuPath software (v0.5.1). Tumor regions were delineated using the polygon tool, while TILs were labeled individually with high-precision point annotation. All annotations underwent review by a third senior pathologist before being saved in JSON format.

During preprocessing, Gaussian filtering (σ=1.5) and median filtering (3×3 kernel) were applied for noise reduction, with missing regions reconstructed using bilinear interpolation. To maintain consistency with training conditions, the same data augmentation strategies were employed during inference, including random horizontal/vertical flipping, 90-degree rotation, and HSV color space perturbations (hue: ± 10%, saturation: ± 15%, value: ± 10%).

#### 2.5.2 Core architecture and training process of the automated annotation model

This automated annotation system integrates three specialized deep learning modules. For tissue contour segmentation, an optimized OpenCV-based image processing pipeline was implemented, where WSIs were first downsampled to 2.5× resolution before tissue boundaries were extracted using the Roberts edge detection operator. Binarization was achieved through Otsu’s adaptive thresholding method, and to enhance weak boundary detection, the parameters of the Canny edge detector were optimized to (30, 120). Two morphological closing operations using a 3×3 elliptical structuring element were applied to fill internal holes while preserving tissue architectural details.

For tumor parenchyma segmentation, a lightweight U²-NetP architecture was employed. This model features a nested U-shaped structure with residual U-blocks that integrate both dilated and standard convolutions. This design expands the receptive field while preserving spatial resolution, enabling it to effectively capture multi-scale features. These features span from local cellular details, such as nuclear morphology and glandular structures, to global tissue organization, which includes gland distribution patterns and stromal fiber orientation.

For lymphocyte identification, the system utilizes the YOLOv7 framework. Its architecture combines an E-ELAN backbone with an FPN+PAN neck for multi-scale feature fusion, enabling the simultaneous capture of local cellular textures and global distribution patterns of lymphocytes.

#### 2.5.3 Experimental environment

All computational experiments were performed on a system equipped with an NVIDIA Tesla A100 40GB GPU and an Intel Xeon Gold 6226R CPU.

#### 2.5.4 Iterative annotation refinement with human-in-the-loop

To construct a high-quality training dataset for this study, we adopted a human-in-the-loop strategy. An initial automated annotation model (19) was first applied to pre-annotate all WSIs. Two experienced pathologists then independently reviewed the automated annotations using QuPath software. They corrected misidentified cells or regions, added missed targets, and removed false positives. The refined annotations were used as the ground truth for subsequent model optimization and immune subtyping analysis.

### 2.6 Development of a morphology-based immune subtype prediction model

An interpretable prediction model was developed to bridge pathological morphology with transcriptome-based immune subtypes. This was achieved by establishing a quantitative pipeline for analyzing spatial distributions of TILs from automated annotations and deriving corresponding immune subtype classification rules.

#### 2.6.1 Quantification of TILs spatial features

WSIs from the 80-case training set were processed using a self-developed automated annotation model (methodological details described in a separate publication under preparation; the model was further optimized with data from the current study). Each WSI was divided into local patches using a sliding window approach with 100 μm × 100 μm step size. Tumor parenchyma and extra tissue regions were filtered out, retaining only stromal areas for TIL counting. The TIL count for each patch was displayed in the upper-left corner of the corresponding sub-image.

To standardize the maximum and minimum TIL counts across different patches and slides, the number of TILs detected in each patch was normalized to a range of 0–1. Here, represents the TIL count in a given patch, denotes the maximum TIL count observed across all patches, and the value represents the relative lymphocyte density of the patch, defined as the ratio of its TIL count to the maximum count. For each WSI, normalized scatter plots were generated based on the-values, as illustrated in **Supplementary Figures 1A–D**. The specific normalization formula applied is as follows:


$$a=\frac{Num}{Num_{Max}}$$

#### 2.6.2 Determination of high-attention region threshold

Through the analysis of the aforementioned scatter plots, we observed a distinct bimodal clustering pattern in the image patches, characterized by a densely populated cluster of low-density patches and a sparser cluster of high-density patches (**Supplementary Figures 1A–D**). Based on this observation, the latter was defined as “High-attention regions” To quantitatively distinguish these two clusters, we calculated the inter-cluster distance using hierarchical clustering algorithms. Distribution analysis on the training set data revealed that the interval with *a*-value > 0.2 exhibited high stability and strong discriminative power (**Supplementary Figure 1E**), leading to the determination of *a* > 0.2 as the optimal threshold for defining high-attention regions. The rationale for selecting this value and detailed validation of its effectiveness is provided in the **Supplementary Materials**.

#### 2.6.3 Formulation of immune subtype classification rule

Based on the proportion of high-attention region patches relative to the total number of valid patches (within tissue contours), a “High-Aggregation TILs Patch Ratio Score” was calculated as the immune score for each WSI. The specific formula is as follows:


$$Score=\frac{N\_high}{N\_valid}$$

Here, *N_high* denotes the number of high-attention region patches, and N_valid​ represents the total number of valid patches within the tissue contour that contain lymphocyte nuclei. A patch is considered valid if the center point of a lymphocyte detection bounding box lies within the tissue contour.

As illustrated in **Supplementary Figure 1F**, performance optimization on the training set established a threshold of Score>0.05 for classifying a sample as “Immunity_H”; otherwise, it is classified as “Immunity_L”.

#### 2.6.4 Creation of TILs spatial distribution heatmaps

To enhance model interpretability, fine-grained heatmaps were generated based on the *a*-value of each image patch. These heatmaps were created using spatial linear interpolation to produce continuous color mapping, intuitively displaying the spatial distribution gradient of TILs within the tumor tissue. In these visualizations, red and blue colors indicate high-attention and low-attention regions, respectively.

### 2.7 Immunohistochemical staining validation and quantitative analysis

IHC analysis was performed on the external validation cohort to validate model-predicted immune subtypes at the protein level. Two senior pathologists (QHZ and LYZ), blinded to sample identities, selected representative formalin-fixed paraffin-embedded tissue blocks from 60 LUAD cases based on the following criteria: (1) presence of both tumor and adjacent normal tissue on the same section; (2) tumor tissue comprising >30% of the sectional area; (3) minimal necrotic or hemorrhagic areas (<5% necrosis). Consecutive sections from qualified blocks were stained for CD3 (pan-T cell marker), CD8 (cytotoxic T lymphocytes), CD20 (B cells), and CD68 (macrophages). Detailed IHC protocols, including antibody specifications and staining procedures, are provided in **Supplementary Table 1**.

All stained sections were digitized using a Motic-Easyscan whole-slide scanner. Two independent pathologists, blinded to clinical and prediction data, quantified immune cell densities by counting CD3^+^, CD8^+^, CD20^+^, and CD68^+^ cells across 10 randomly selected 400×fields per sample. After excluding the highest and lowest counts, the mean of the remaining fields served as the final quantitative measure for statistical analysis.

To further assess the biological relevance of the model−predicted immune subtypes, these IHC data were used to stratify the 60 external cohort cases into high− and low−immunity groups. For each case, the mean counts per high−power field (HPF; 0.2595 mm²) for CD3, CD8, CD20, and CD68 were converted to cell densities (cells/mm²). Densities for each marker were Z−score normalized across all 60 cases, and a composite IHC score was calculated as the average of the four Z−scores:


$$\text{IHC }{\text{score}}_{i}=\frac{Z_{i,\text{CD}3}+Z_{i,\text{CD}8}+Z_{i,\text{CD}20}+Z_{i,\text{CD}68}}{4}$$

Cases were dichotomized into IHC−high and IHC−low immunity groups based on the median composite score of the cohort. This median−based stratification provided a balanced reference for evaluating whether the model−predicted subtypes reflect biologically meaningful differences in immune cell infiltration.

### 2.8 Automated annotation model performance evaluation

Model performance was assessed using task-specific metrics. For tissue contour detection and tumor parenchyma segmentation, the Dice Similarity Coefficient (DSC), precision, and recall were employed. Lymphocyte identification performance (based on YOLOv7) was evaluated using precision, recall, F1-score and mAP@0.5. The immune subtype prediction model was validated by receiver operating characteristic (ROC) analysis, with the area under the curve (AUC) quantifying classification accuracy in the internal validation cohorts.

### 2.9 Statistical analysis

Continuous variables were tested for normality and are presented as mean ± standard deviation if normally distributed, compared using the independent samples t-test. Categorical variables are expressed as numbers and compared using the chi-square test. All analyses were performed with SPSS 25.0 and R 4.5.0, with two-sided p-values < 0.05 considered statistically significant.

### 2.10 Study flowchart

**Figure 1:**
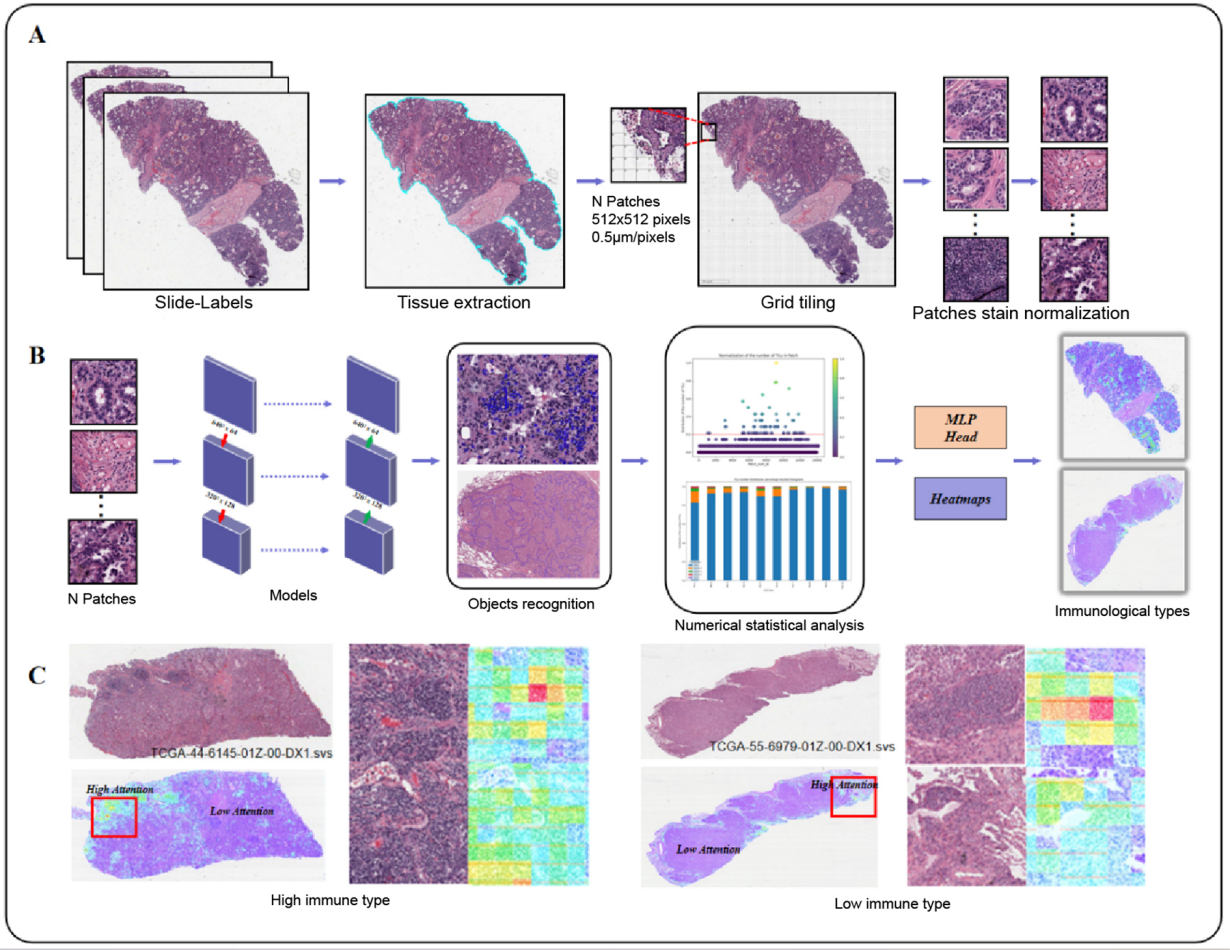
Flowchart of immune subtyping modeling. **(A)** Data preprocessing workflow in intelligent recognition model construction. **(B)** Development of intelligent recognition model and immune subtyping model. **(C)** Visualization of subtyping results through heatmaps for high and low immune groups.

The overall workflow of this study is illustrated in **Figure 1**, which outlines the key steps from data preprocessing and model development to validation and interpretation.

## 3 Results

### 3.1 Construction of immune subtypes based on transcriptomic data and biological rationality analysis

#### 3.1.1 Construction of immune subtypes using transcriptomic data

**Figure 2:**
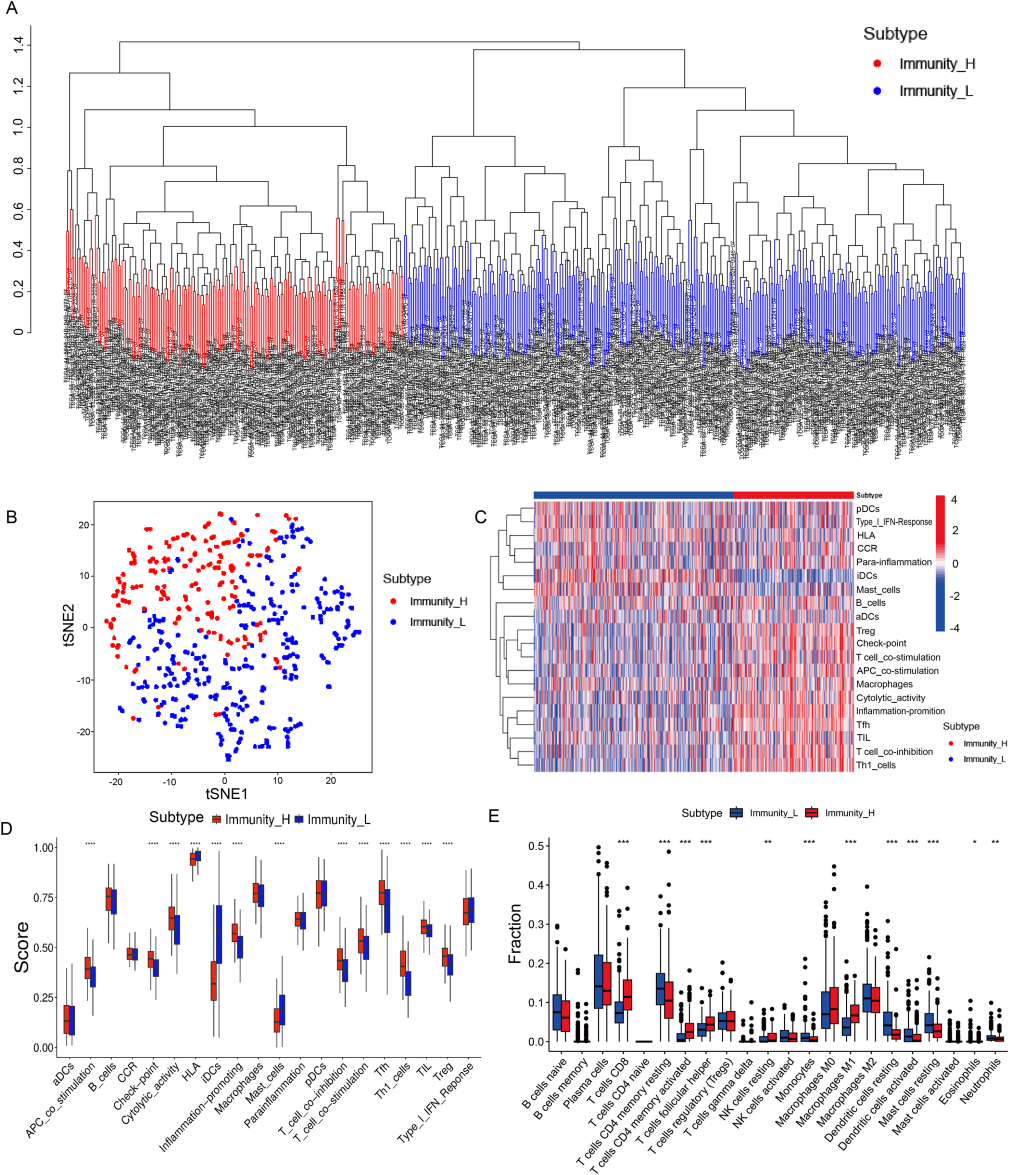
Transcriptomic immune subtyping and functional characterization of the LUAD cohort. **(A)** Clustering analysis categorized patients into two groups: high immune group and low immune group. **(B)** t-SNE plot visualizing the classification results of cluster analysis. **(C)** Heatmap showing a coordinated upregulation of immune functions in the high-immunity subgroup. **(D)** Box plot confirming significant activation of key immune processes in the high-immunity subgroup, in contrast to elevated immature DC and HLA scores in the low-immunity subgroup. **(E)** Differential infiltration of 22 immune cell types between immunity groups. Cell proportions were estimated by CIBERSORT. * indicates *p* < 0.05, ** indicates *p* < 0.01 and *** indicates *p* < 0.001.

Hierarchical clustering analysis of ssGSEA-derived immune signature enrichment scores classified the 503 patients into two distinct subtypes. The dendrogram was cut at the point that yielded the most balanced bifurcation based on its structure, resulting in a high-immunity group (189 patients) and a low-immunity group (314 patients) (**Figure 2A**). This classification exhibits a clear separation pattern in the t-SNE reduced-dimensional space, confirming its intrinsic heterogeneity and classification validity (**Figure 2B**). The clustering heatmap generated based on ssGSEA immune scores (**Figure 2C**) shows that the high-immunity group exhibits synergistic and widespread activation across the vast majority of immune-related pathways, whereas the low-immunity group demonstrates a general suppression of corresponding immune functions compared to the high-immunity group. To further quantify these differences, we directly compared 20 core immune functions represented in the heatmap. The results demonstrated that the high-immunity group had significantly higher scores across multiple immune functions, including APC co-stimulation, immune checkpoints, cytolytic activity, inflammation promotion, T-cell co-stimulation, Tfh cells, Th1 cells, TIL and Treg. These findings functionally confirm that adaptive immune responses in the high-immunity group are broadly activated across multiple core processes, including antigen presentation, T-cell activation and co-stimulation, cytotoxic killing, and immune regulation. In contrast, the low-immunity group showed higher scores in immature dendritic cells (iDCs) and HLA-related functions, which may suggest defects in antigen presentation or a resting, unactivated state in this group (**Figure 2D**).

#### 3.1.2 Analysis of immune cell composition differences between immune subgroups

To validate the biological relevance of the identified immune subtypes, immune cell composition was analyzed using the CIBERSORT algorithm. Quantitative analysis of 22 immune cell types revealed that the high-immunity group showed significant enrichment of key anti-tumor effector cells, including CD8^+^ T cells, activated memory CD4^+^ T cells, follicular helper T cells, and M1 macrophages (**Figure 2E**). In contrast, the low-immunity group was predominantly characterized by immunosuppressive or quiescent immune cells, such as resting memory CD4^+^ T cells, monocytes, and resting dendritic cells. These distinct cellular profiles confirm that the transcriptome-defined immune subtypes represent two fundamentally different states of the tumor immune microenvironment, characterized by either immune activation or immune suppression.

#### 3.1.3 Genomic characteristics and prognostic associations

**Figure 3:**
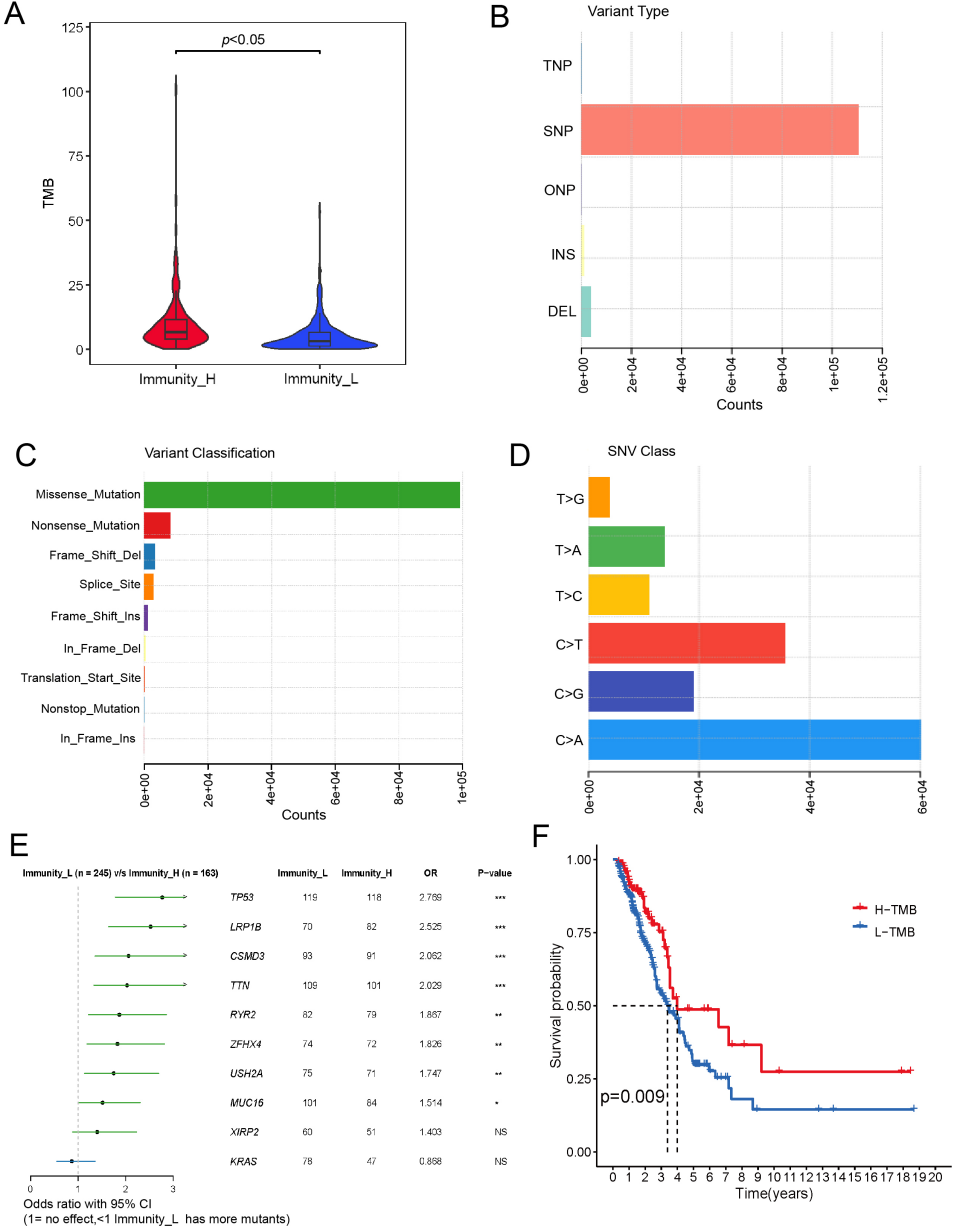
Comparison of genomic alteration profiles between transcriptome-defined high- and low-immune subgroups in LUAD. **(A)** Differences in TMB between the high- and low-immune subgroups. **(B)** The distribution of variant types identified in the cohort, showing single nucleotide polymorphisms as the predominant class. **(C)** Variant classification. **(D)** Distribution of single-nucleotide variant classes in the sample. with “>“ indicating the change from reference to variant base. **(E)** Forest plot of odds ratios for mutated genes between high and low immunity groups. **(F)** Kaplan-Meier survival curves for patients in the high and low TMB groups.

Genomic differences between immune subtypes were investigated through comparative analysis. TMB was significantly higher in the high-immunity group (*p* < 0.001), suggesting that elevated mutation load may reshape the immune microenvironment through neoantigen-induced immune activation (**Figure 3A**). Analysis of mutation types across all samples identified missense mutations, nonsense mutations, frameshift deletions, splice site mutations, and frameshift insertions as the five most frequent categories (**Figure 3B**). Single nucleotide polymorphisms represented the predominant variant type, with C>A as the most common base substitution (**Figures 3C, D**).

Differential gene mutation analysis revealed TP53, TTN, MUC16, CSMD3, and LRP1B as the five genes showing the most significant mutation frequency differences between subtypes, all exhibiting higher mutation rates in the high-immunity group (**Figure 3E**). Prognostic analysis demonstrated significantly better overall survival in high-TMB patients compared to low-TMB patients (p < 0.05, **Figure 3F**). These genomic findings provide multi-faceted support for the biological validity of the immune subtyping system.

#### 3.1.4 Analysis of transcriptomic differences and pathway characteristics

**Figure 4:**
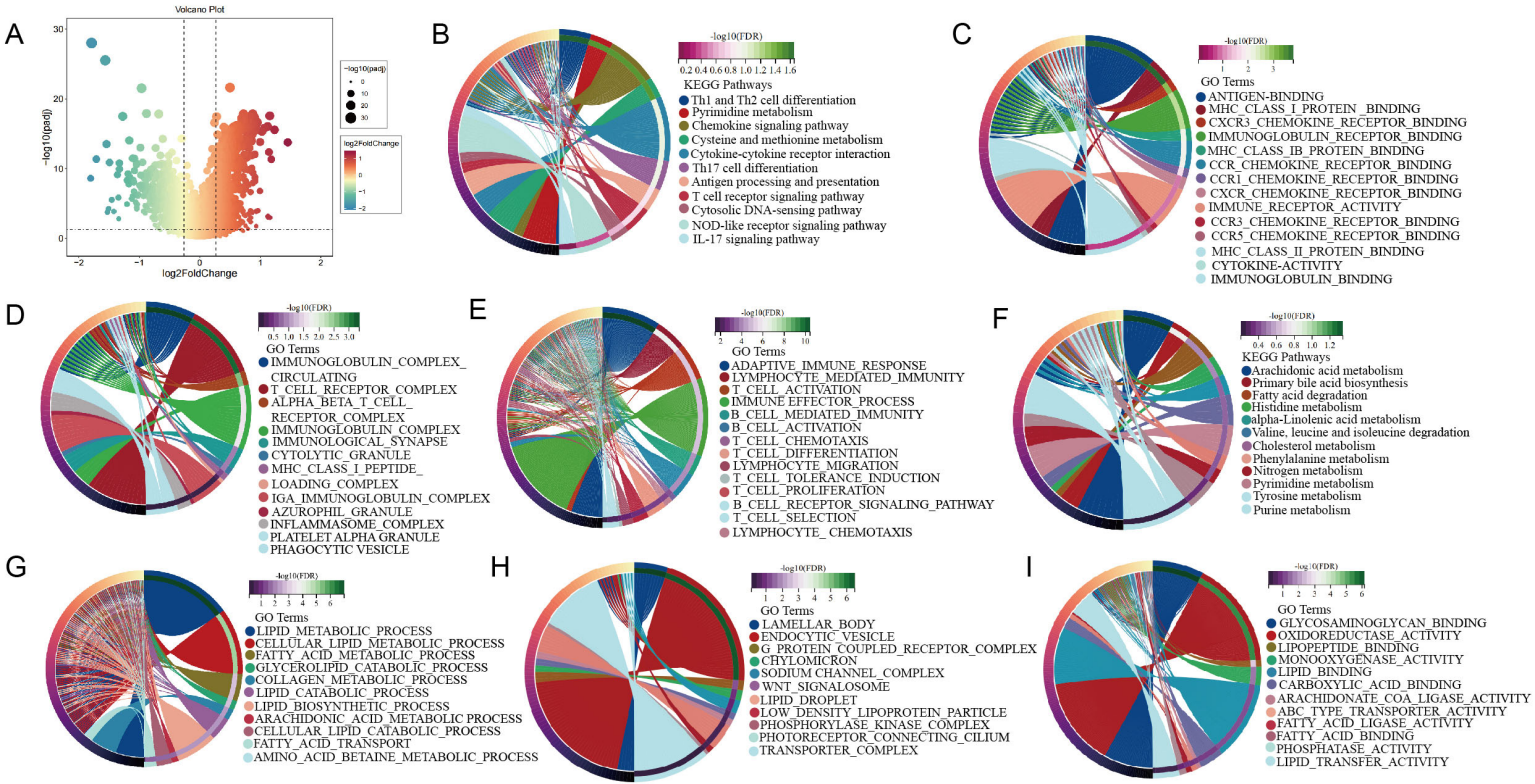
Enrichment analysis results of differentially expressed genes in the high- and low-immune group. **(A)** Volcano plot showing 2,194 differentially expressed genes. **(B)** KEGG pathways enriched in the high-immunity group, primarily immune-related. **(C–E)** GO analysis of the high-immunity group: molecular functions **(C)**, cellular components **(D)**, and biological processes **(E, F)** KEGG pathways enriched in the low-immunity group, primarily metabolic. **(G–I)** GO analysis of the low-immunity group: biological processes **(G)**, cellular components **(H)**, and molecular functions **(I)**.

Using thresholds of |log_2_FC| ≥ 1.2 and FDR < 0.05, differential expression analysis identified 2,194 genes distinguishing the high- and low-immunity groups (**Figure 4A**). The complete list of these differentially expressed genes is provided in **Supplementary Table 2**. KEGG pathway enrichment revealed that upregulated genes in the high-immunity group were significantly enriched in immune-related pathways including Th1/Th2 cell differentiation, antigen processing and presentation, T cell receptor signaling, cytokine-cytokine receptor interaction, and NOD-like receptor signaling (**Figure 4B**). Conversely, upregulated genes in the low-immunity group showed primary enrichment in tumor metabolic pathways such as pyrimidine metabolism and cysteine-methionine metabolism (**Figure 4F**).

GO analysis systematically characterized subtype-specific functional patterns. In the high-immunity group, biological processes were dominated by adaptive immune response, lymphocyte activation and regulation, and immune cell chemotaxis (**Figure 4E**). Cellular components included immune protein complexes, receptor complexes, and specialized immune cell structures (**Figure 4D**), while molecular functions centered on antigen binding, immunoglobulin receptor binding, and chemokine receptor activities (**Figure 4C**). The complete lists of differentially expressed genes and the corresponding GO enrichment results are provided in **Supplementary Table 3**.

The low-immunity group exhibited distinct functional profiles, with biological processes enriched in lipid metabolism, fatty acid metabolism, and lipid biosynthesis (**Figure 4G**). Cellular components featured lipid storage structures, lipoprotein transport complexes, and cellular signaling apparatus (**Figure 4H**), and molecular functions emphasized lipid binding, oxidoreductase and ligase activities, and transmembrane transport functions (**Figure 4I**).

### 3.2 Iterative optimization and performance validation of the automated annotation model

#### 3.2.1 Construction of a high-quality annotated dataset

An automated annotation model previously developed by our team (19) was employed to process H&E-stained digital pathology images. This model, originally trained on a high-quality LUAD dataset containing over 20,000 annotated units, automatically generates annotations for tissue contours, tumor parenchyma, and lymphocytes. For the current study, the dataset was expanded with more than 10,000 additional annotated units, including 28 tissue contour annotations, 1,352 tumor parenchyma annotations, and 8,763 TIL annotations, to iteratively optimize model performance.

#### 3.2.2 Performance validation of the optimized automated annotation model

The original model was retrained using supplementary annotated data from this study, producing a performance-enhanced iterative version. This enhanced model was then applied to automatically annotate all WSIs. All automated results underwent comprehensive manual review and refinement by an experienced pathologist to ensure annotation quality before being used for predictive model construction.

**Figure 5:**
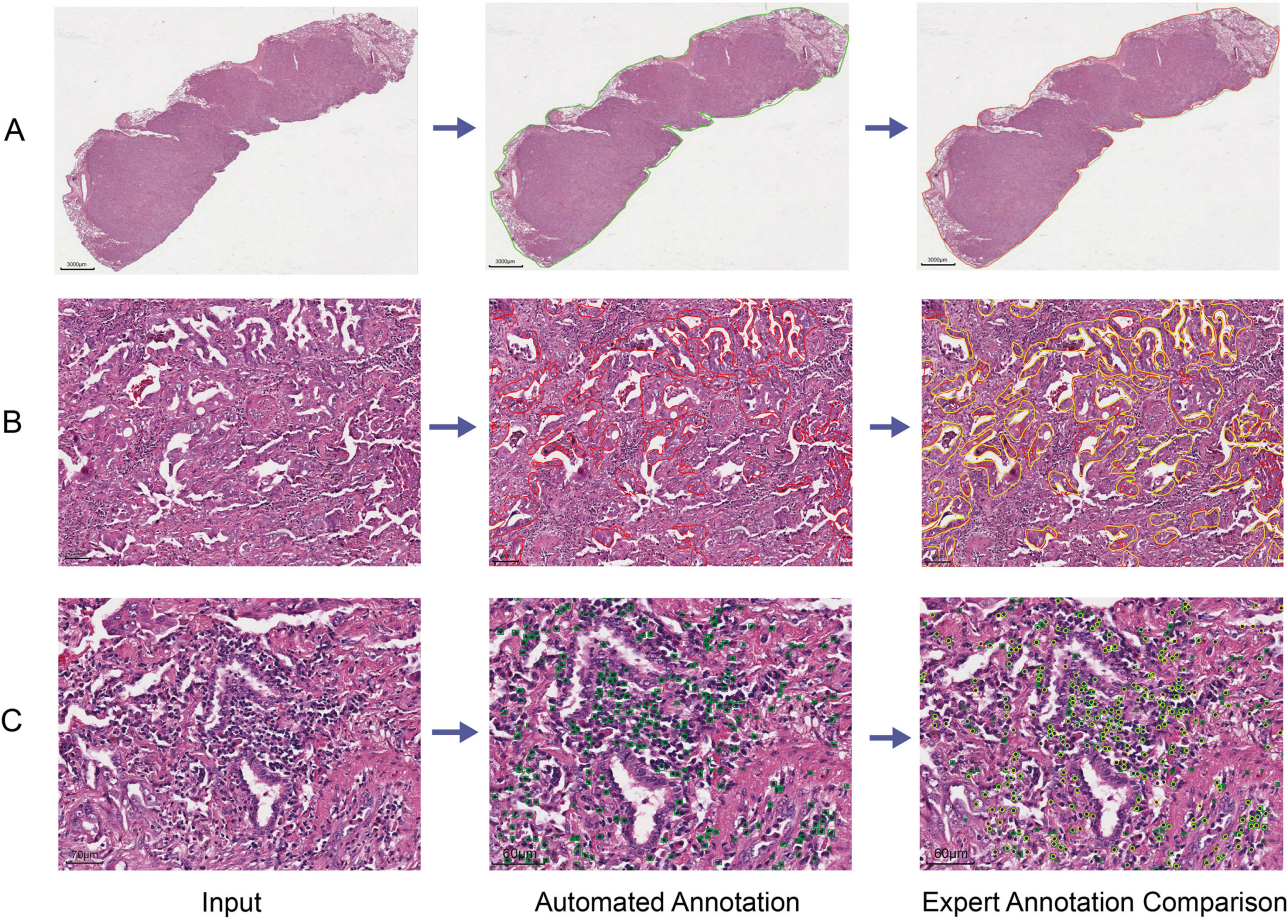
Comparison of model’s identification and annotation results with manual annotations across tasks. **(A)** Tissue contour recognition. The left panel shows the original WSI. The middle panel shows the tissue contour automatically annotated by the model (green outline). The right panel presents a side-by-side comparison of manual annotation (red outline) and model annotation (green outline) on the same tissue image. **(B)** Tumor parenchyma segmentation. The left panel shows the original WSI. The middle panel shows the tumor parenchyma automatically segmented by the model (red overlay). The right panel presents a side-by-side comparison of manual annotation (yellow overlay) and model segmentation (red overlay) on the same tissue image. **(C)** Lymphocyte identification. The left panel shows the original WSI. The middle panel shows lymphocytes automatically detected by the model (green boxes). The right panel presents a side-by-side comparison of manual annotation (yellow circles) and model detection (green boxes) on the same tissue image. All manual annotations shown here are the ground truth labels used for model training, which were manually annotated and verified by experienced pathologists.

The optimized model demonstrated excellent performance on the test set. The tissue contour detection model showed improved capability in identifying complex structures, achieving precision of 93.94%, recall of 96.47%, and Dice coefficient of 95.09% (**Figure 5A**, **Table 1**), establishing a reliable spatial foundation for subsequent analyses. For tumor parenchyma segmentation, the enhanced model performed better in regions with ambiguous tumor-stroma boundaries, attaining precision of 90.69%, recall of 90.57%, and Dice coefficient of 91.53% (**Figure 5B**, **Table 1**). In the particularly challenging task of lymphocyte detection, model sensitivity was significantly improved through targeted training data optimization, especially in high-density lymphocyte regions. The final model achieved a precision of 78.94%, recall of 80.12%, F1-score of 79.51% and mAP@0.5 82.13% (**Figure 5C**, **Table 1**). Collectively, this high-performance automated pipeline provided the essential technical foundation for large-scale image analysis throughout this study.

**Table 1:** Performance evaluation of the model.

Task	Model type	DICE coefficient	F1-Score	mAP@0.5	Precision	Recall
Tumor parenchyma segmentation	U^2^-NetP	91.53%	\	\	90.69%	90.57%
Tissue contour detection	OpenCV-based pipeline	95.09%	\	\	93.94%	96.47%
Lymphocyte detection	YOLOv7	\	79.51%	82.13%	78.94%	80.12%

On this basis, we supplemented a small amount of manually annotated data, thereby obtaining 80 cases of LUAD -WSIs with fully annotated TILs as the foundation for subsequent modeling.

### 3.3 Development of a morphology-based immune subtype prediction model and multi-level performance validation

#### 3.3.1 Development and preliminary validation of the immune phenotyping prediction model

Based on automated annotations derived from 80 training WSIs, which were all manually corrected and supplemented by experienced pathologists following initial automated annotation, this study successfully developed an interpretable immune phenotyping prediction model centered on the spatial distribution patterns of TILs. The model’s core innovation lies in translating complex lymphocyte spatial arrangements into a concise, transparent binary classification rule.

First, by analyzing the spatial density distribution of TILs, we defined a “high-attention region” to characterize areas of lymphocyte aggregation. Through normalization and cluster analysis of TIL density across all image patches, patches with normalized values exceeding 0.2 consistently corresponded to regions of focal, dense lymphocyte infiltration, thereby clearly delineating them as high-attention regions.

**Figure 6:**
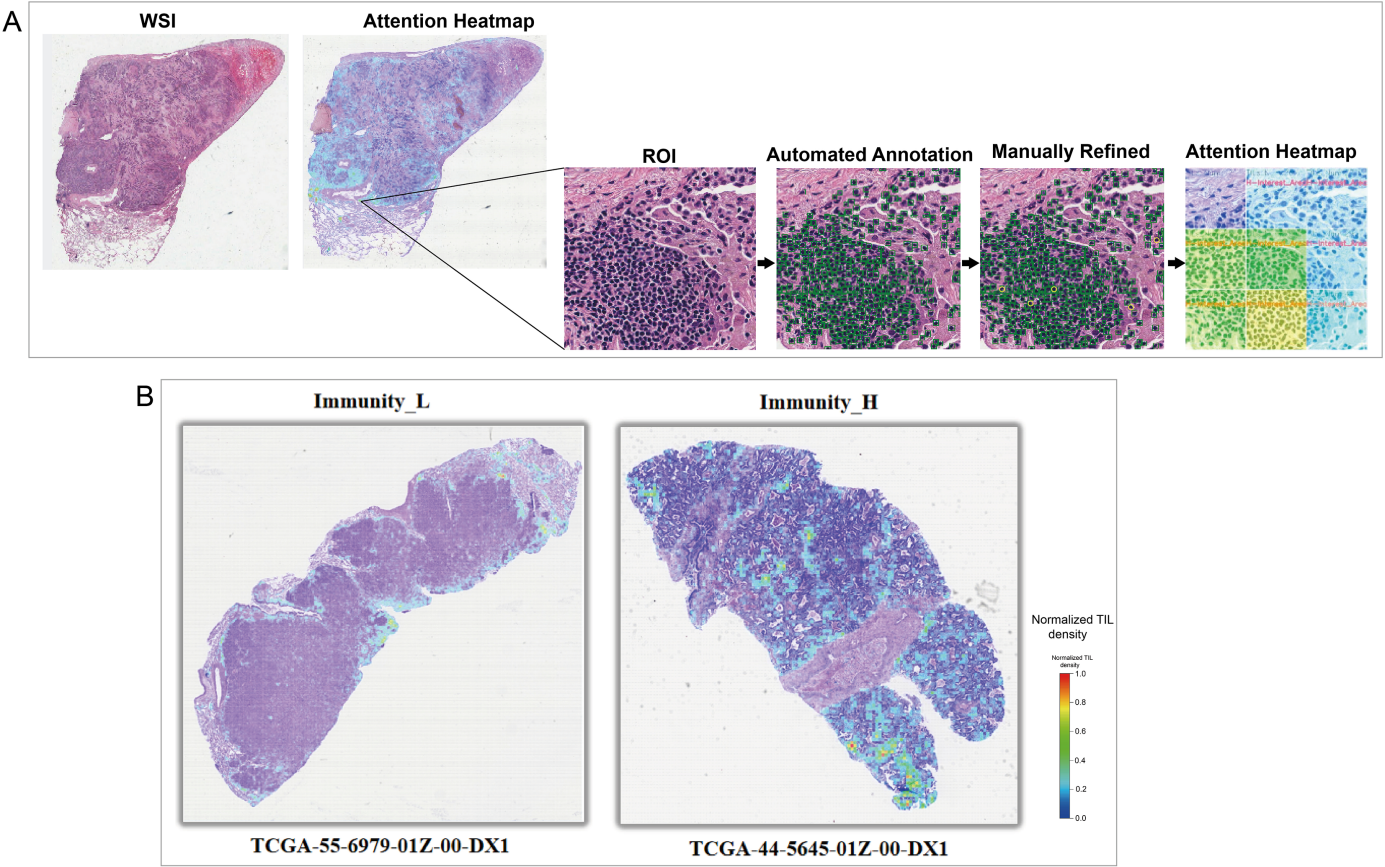
Generation and visualization of TIL spatial distribution heatmaps. **(A)** Schematic pipeline illustrating the workflow for generating attention heatmaps of TIL spatial distribution in the internal validation cohort. The process begins with H&E-stained WSIs, followed by zoomed ROI, automated TIL annotation, and final attention heatmap overlays. Green boxes represent the results of automated annotation by the pipeline. **(B)** Representative attention heatmaps from the internal validation cohort, showing distinct spatial patterns of TIL distribution between samples classified as high-immunity and low-immunity by the model. Red regions indicate areas with dense TIL infiltration, while blue regions represent areas with sparse or absent TIL infiltration.

Subsequently, based on this quantitative assessment of TIL spatial distribution, the final decision rule for immune phenotyping was established. Analysis indicated that the proportion of high-attention regions across the entire slide served as the key discriminating feature for overall immune status. After threshold optimization within the model development cohort, the classification rule was finalized whereby samples are classified into the high-immunity group (Immunity_H) if the proportion of high-attention regions exceeds 0.05; otherwise, they are assigned to the low-immunity group (Immunity_L). This rule effectively translates morphological spatial features into interpretable immune phenotypes. Fine-grained heatmaps visually illustrate that cases classified as high-immunity display extensive and dense TIL high-attention regions throughout the tissue, whereas such regions are markedly reduced in low-immunity cases (**Figure 6**).

To preliminarily evaluate the model’s performance, we randomly selected 10 cases from the internal validation cohort (4 high-immunity, 6 low-immunity, as defined by transcriptomic analysis) as a small-sample test set. The model achieved a classification accuracy of 90% on this test set (**Table 2**). The single misclassified case had a high-attention region proportion of 0.082, exceeding the 0.05 threshold, but was transcriptomically classified as low-immunity, suggesting potential tumor heterogeneity or borderline immune status. These initial findings demonstrate satisfactory classification performance, supporting the need for more comprehensive internal and external validation.

**Table 2:** Immune subtype predictions of the model on the test set.

TCGA-ID	Proportion of high−attention regions	True label	Predicted results
TCGA-55-6979-01Z-00-DX1	0.025260	Immunity_L	Immunity_L
TCGA-55-6975-01Z-00-DX1	0.015157	Immunity_L	Immunity_L
TCGA-55-6972-01Z-00-DX1	0.005871	Immunity_L	Immunity_L
TCGA-44-5645-01Z-00-DX1	0.058781	Immunity_H	Immunity_H
TCGA-44-6778-01Z-00-DX1	0.053232	Immunity_H	Immunity_H
TCGA-44-2665-01Z-00-DX1	0.042932	Immunity_L	Immunity_L
TCGA-44-3398-01Z-00-DX1	0.034461	Immunity_L	Immunity_L
TCGA-44-2661-01Z-00-DX1	0.082299	Immunity_H	**Immunity_L**
TCGA-49-4490-01Z-00-DX1	0.020078	Immunity_L	Immunity_L
TCGA-44-6145-01Z-00-DX1	0.064477	Immunity_H	Immunity_H

Bold indicates the misclassified case.

#### 3.3.2 Internal validation of the immune phenotyping model

**Figure 7:**
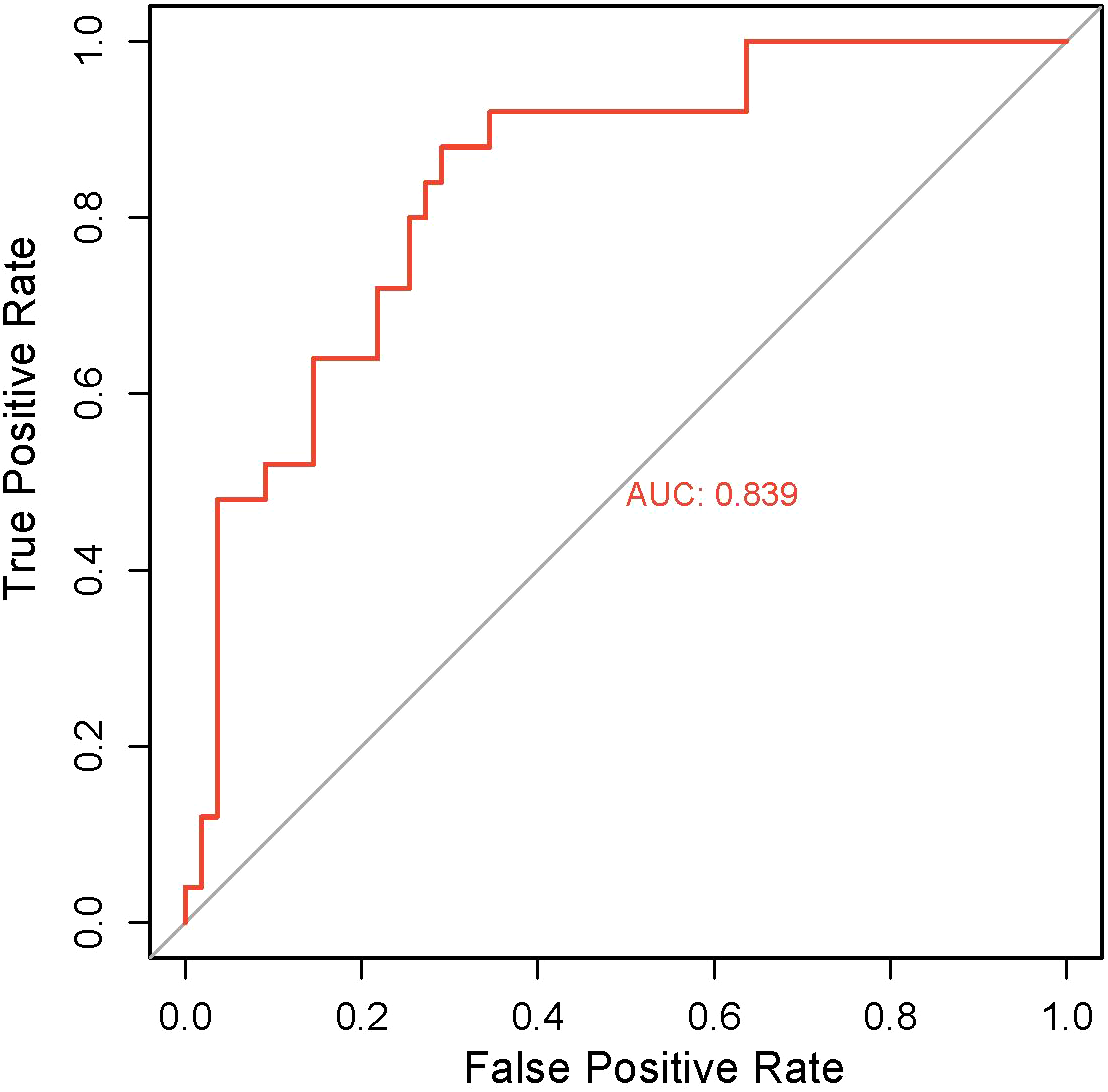
ROC curve of the immune type classification model.

**Figure 8:**
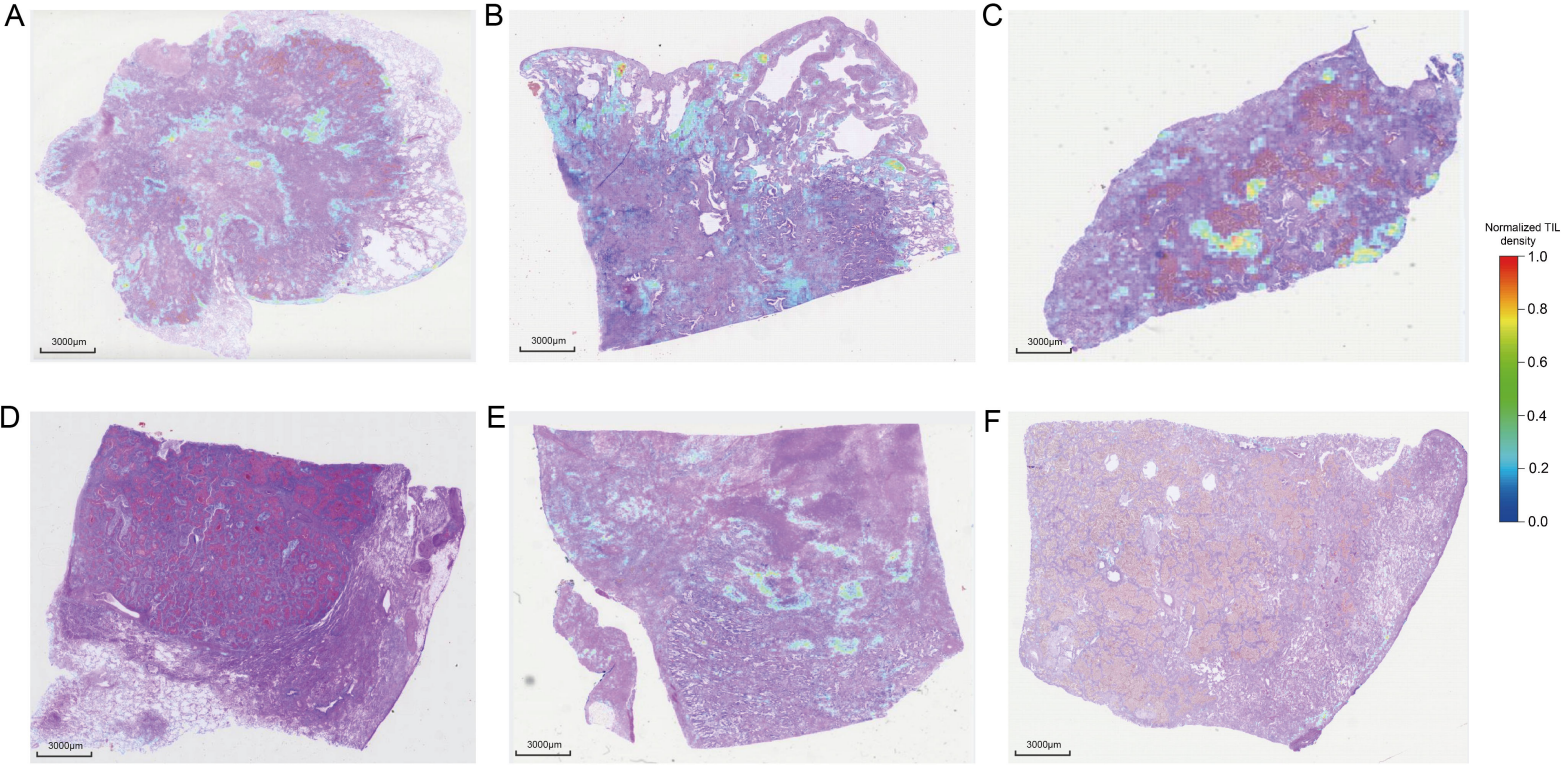
TILs spatial distribution heatmaps of high- and low-immune subgroups in the internal validation cohort. **(A–C)** Three representative cases predicted as the high-immune subgroup. **(D–F)** Three representative cases predicted as the low-immune subgroup. Color bar indicates normalized TIL density, ranging from 0 (blue, sparse or absent infiltration) to 1 (red, dense infiltration).

To evaluate the robustness of the immune phenotype prediction model in independent samples, we performed systematic validation using an internal cohort of 100 WSIs. The automated annotation pipeline developed in this study was first applied to conduct fully automated analysis of all slides, generating standardized spatial distribution data for TILs. These data were subsequently input into the prediction model to obtain immune phenotype classifications for each sample. Prediction accuracy was assessed by comparing model outputs with transcriptome-based immune phenotypes for the cohort (clinical baseline characteristics provided in **Table 3**). ROC curve analysis revealed an AUC of 0.839 (**Figure 7**), indicating strong classification performance. Furthermore, fine-grained heatmaps generated based on the automated annotation results visually demonstrate the differences in TILs spatial distribution between high- and low-immunity group cases (**Figure 8**), providing visual evidence to support the prediction outcomes.

**Table 3:** Clinical baseline characteristics of the included cases.

Clinical characteristic	Training cohort(n=80)	Internal validation cohort(n=100)	External validation cohort(n=60)	*p*
Age, mean ± SD	66.04 ± 10.31	64.39 ± 10.34	58.88 ± 8.01	< 0.05
Sex, n (%)				0.585
Male	41 (51.3%)	49 (49.0%)	26 (43.3%)	
Female	39 (48.8%)	51 (51.0%)	34 (56.7%)	
TNM Stage Group, n (%)				0.744
Stage I	42(52.5%)	64(64.0%)	34 (56.7%)	
Stage II	21(26.2%)	20(20.0%)	14 (23.3%)	
Stage III	14(17.5%)	11(11.0%)	10 (16.7%)	
Stage IV	3(3.8%)	6(6.0%)	2 (3.3%)	

#### 3.3.3 Genomic-level internal validation of model predictions

**Figure 9:**
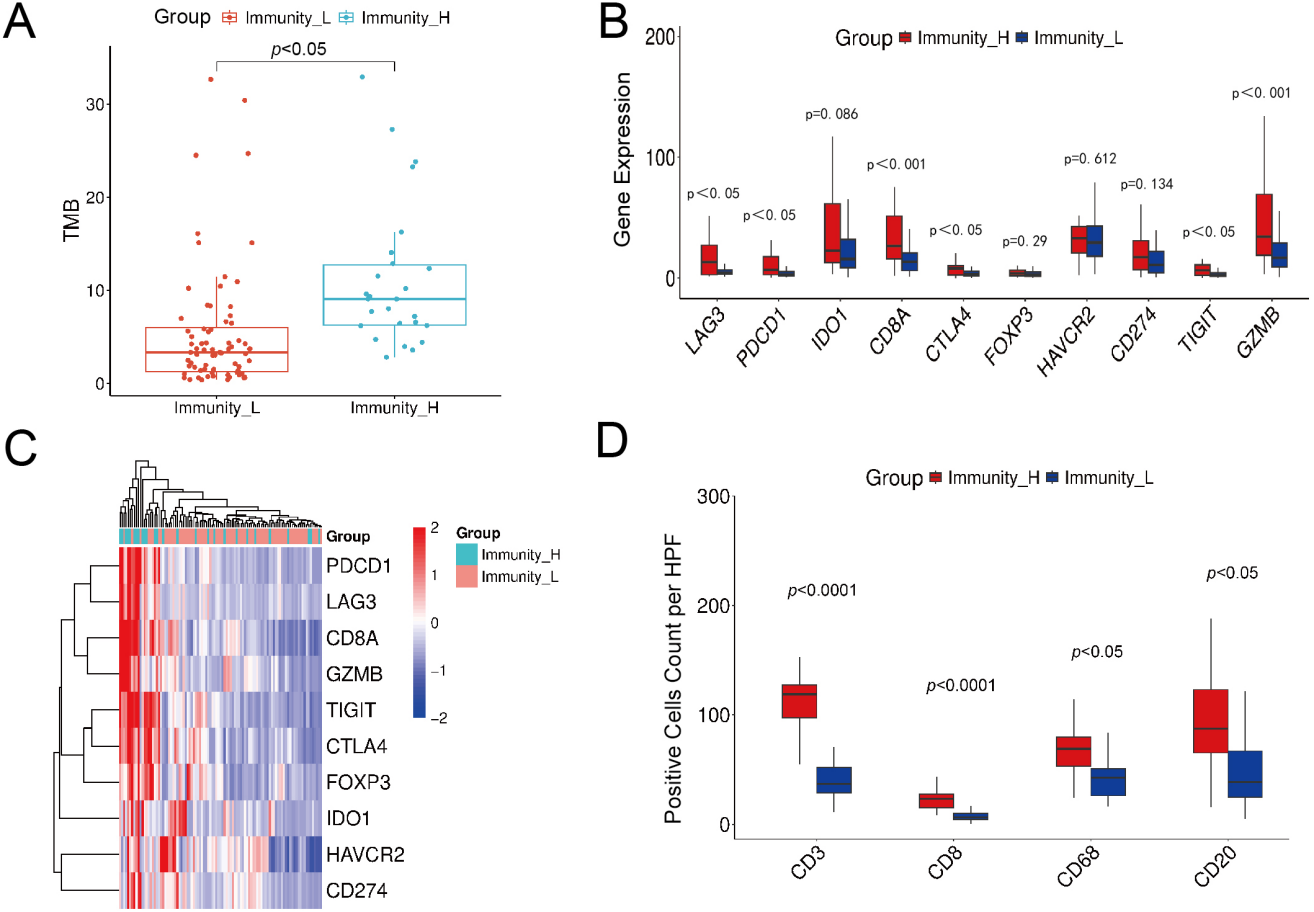
Molecular validation of predicted immune subtypes. **(A)** Analysis of TMB differences between model-predicted high and low immune subgroups. **(B)** The box plot illustrates the expression differences of 10 immunotherapy-related genes across distinct immune subtype groups. **(C)** Heatmap of immune-related gene expression profiles. **(D)** Comparison of positive cell counts for four immune markers between high- and low-immunity groups.

To validate the biological plausibility of the prediction results at the molecular level, the difference in TMB between the model-predicted high- and low-immunity groups was first analyzed at the genomic level. The results showed that the TMB level in the high-immunity group was significantly higher than that in the low-immunity group (*p* < 0.05). This finding aligns with the known mechanism that high TMB is associated with increased neoantigen production and enhanced immune cell infiltration. Moreover, this observation is consistent with the TMB levels noted between the transcriptome-label-derived high- and low-immunity groups in this study, further confirming that the immune phenotypes identified by our prediction model exhibit reliable intrinsic consistency at the genomic level (**Figure 9A**).

#### 3.3.4 Transcriptome-level internal validation of model predictions

We further evaluated the biological validity of the immune subtyping by examining the expression patterns of 10 core immune-therapy-related genes (CD8A, GZMB, PDCD1, CTLA4, LAG3, TIGIT, FOXP3, HAVCR2, CD274, and IDO1) in the internal validation cohort. Quantitative analysis demonstrated significantly elevated expression of multiple key genes in the high-immunity group, including T-cell marker CD8A, effector molecule GZMB, and immune checkpoint molecules PDCD1 (PD-1), LAG3, CTLA4, and TIGIT (**Figure 9B**). Furthermore, heatmap visualization revealed a distinct expression pattern across all 10 genes, with the high-immunity group showing consistently elevated expression and the low-immunity group exhibiting broadly suppressed levels (**Figure 9C**).

This confirms that our prediction model effectively distinguishes tumor microenvironment subtypes with fundamentally different immune activities, and suggests that patients classified into the high-immunity group may demonstrate enhanced responsiveness to immune checkpoint inhibition therapy.

#### 3.3.5 External validation based on model prediction

To assess the generalizability of the immune subtype prediction model established in this study, we evaluated it on an independent cohort of 60 LUAD cases (see Methods for cohort construction details). The model classified these cases into 28 high-immunity and 32 low-immunity samples based on the distribution of TILs in the WSIs. Clinical baseline characteristics are presented in **Table 3**.

**Figure 10:**
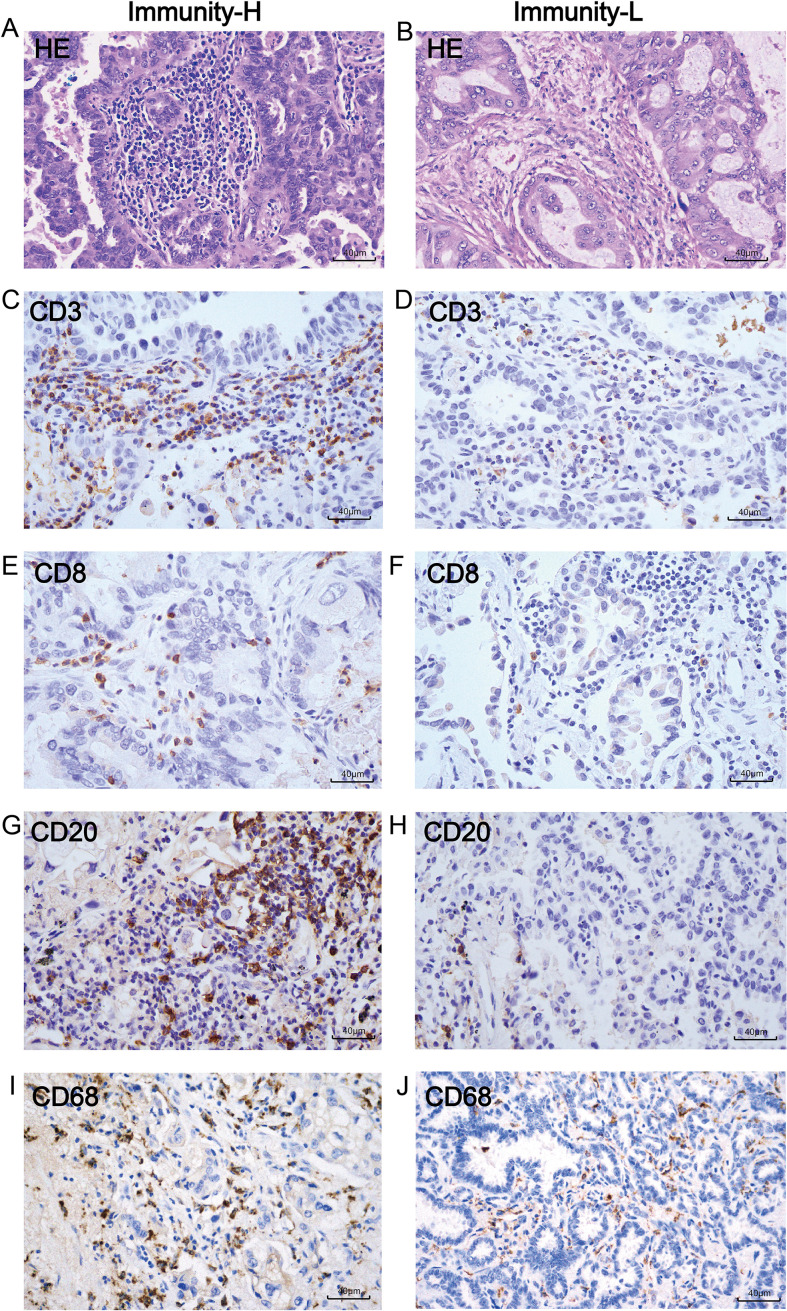
Morphological features of H&E staining and multi-marker immunohistochemical staining in high-immunity and low-immunity groups under microscopy. **(A, B)** H&E images of cases from high- and low-immunity groups at 400× magnification (10×40). **(C, D)** Expression of CD3 in high- and low-immunity groups. **(E, F)** Expression of CD8 in high- and low-immunity groups. **(G, H)** Expression of CD20 in high- and low-immunity groups. **(I, J)** Expression of CD68 in high- and low-immunity groups.

IHC analysis demonstrated significantly higher mean positive cell counts of CD3^+^, CD8^+^, CD20^+^, and CD68^+^ cells in the predicted high- immunity group compared to the low- immunity group (**Figure 9D**, **Table 4**). Histological examination under high magnification revealed extensive lymphocyte infiltration in H&E-stained sections from high-immunity cases, presenting as either diffuse stromal infiltration or focal aggregates (**Figure 10A**). Corresponding IHC-staining confirmed dense infiltration of CD3^+^ T cells and CD8^+^ cytotoxic T cells within both the tumor core and invasive margin (**Figures 10C, E**). CD20^+^ B cells were frequently organized into distinct lymphoid aggregates (**Figure 10G**), while CD68^+^ macrophages showed widespread tissue distribution (**Figure 10I**).

**Table 4:** Comparison of immunohistochemistry-positive cell counts between high- and low-immunity groups.

Marker	Group	N	Median positive cell count [Mean ± SD/Median (IQR)]	*p*
CD3	Immunity-H	29	112.84 ± 28.76	< 0.0001
	Immunity-L	31	44.98 ± 23.45	
CD8	Immunity-H	29	22.98 ± 12.67	< 0.0001
	Immunity-L	31	8.56 ± 7.89	
CD20	Immunity-H	29	83.25 (69.63-114.50)	0.0083
	Immunity-L	31	37.00 (23.75-59.50)	
CD68	Immunity-H	29	61.28 ± 24.56	0.0002
	Immunity-L	31	40.89 ± 22.13	

In contrast, H&E-stained sections from low-immunity cases displayed markedly sparse lymphocyte infiltration (**Figure 10B**). Immunohistochemical analysis corroborated these findings, showing significantly reduced numbers of CD3^+^, CD8^+^, CD20^+^, and CD68^+^ cells with only scattered positive signals detected (**Figures 10D, F, H, J**).

Together, these results provide convergent evidence at both protein expression and histomorphological levels, confirming that model predictions accurately reflect actual immune cell infiltration patterns. The findings fully support the strong generalizability and reliable predictive performance of our immune subtype prediction model.

#### 3.3.6 Quantitative assessment of model performance against IHC-based reference

**Figure 11:**
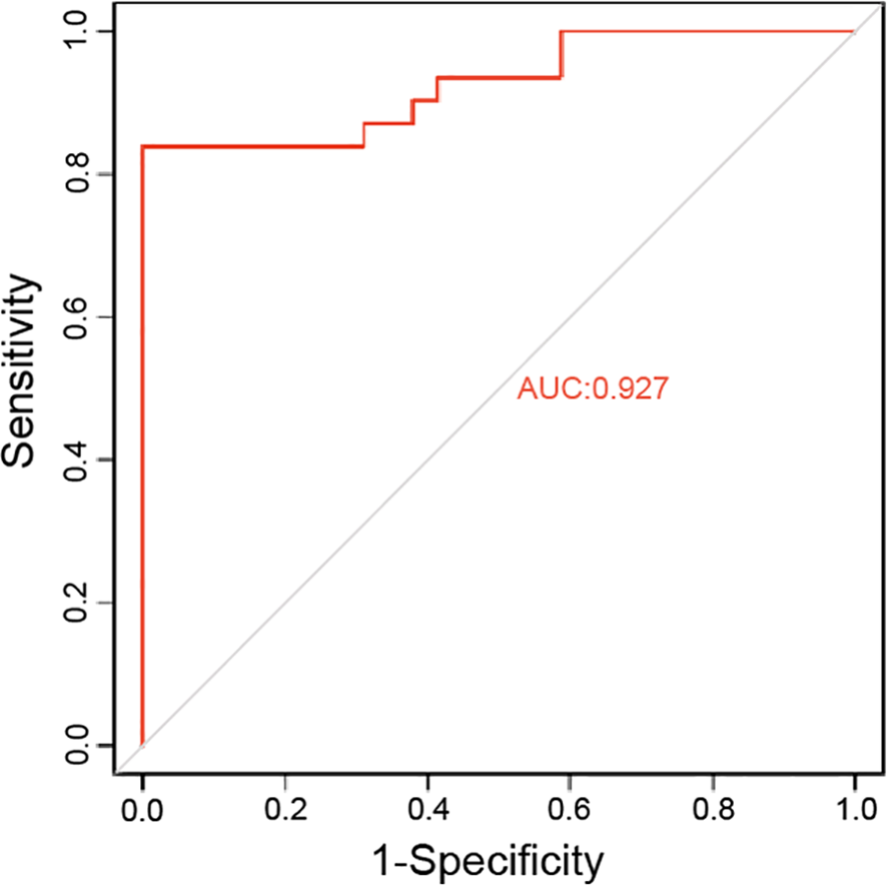
ROC curve of the immune subtype prediction model evaluated against the IHC-based reference. The dashed diagonal line indicates random classification (AUC = 0.5).

To further assess the classification performance of the model in an independent cohort, we compared its predictions against an IHC-based reference in 60 external validation cases. Using the IHC−based reference defined in Methods 2.7 (median−based dichotomization of composite Z−scores from CD3, CD8, CD20, and CD68 densities), the model achieved an accuracy of 90.0% (95% CI: 79.9%–95.3%), with a sensitivity of 86.7% (95% CI: 70.3%–94.7%) and a specificity of 93.3% (95% CI: 78.7%–98.2%). The positive predictive value was 92.9% (95% CI: 76.5%–98.1%), and the negative predictive value was 87.5% (95% CI: 71.9%–95.0%)(**Table 5**). The confusion matrix revealed 26 true positives, 28 true negatives, 2 false positives, and 4 false negatives (**Table 6**). The AUC was 0.927 (95% CI: 0.88–0.98) (**Figure 11**), indicating excellent classification performance of the model in distinguishing between high- and low-immunity phenotypes. These results further confirm the robustness of the model in an independent external cohort.

**Table 5:** External validation performance of the immune subtype prediction model against IHC-based reference.

Metric	Value (%)	95% CI
Accuracy	90.0	79.9 – 95.3
Sensitivity	86.7	70.3 – 94.7
Specificity	93.3	78.7 – 98.2
Positive Predictive Value	92.9	76.5 – 98.1
Negative Predictive Value	87.5	71.9 – 95.0
AUC	0.927	0.88 – 0.98

**Table 6:** Confusion matrix.

	IHC-High	IHC-Low
Model Predicted High	26	2
Model Predicted Low	4	28

## 4 Discussion

The classification of “cold tumors” and “hot tumors” based on the extent and spatial distribution of immune cell infiltration, offers a novel perspective for interpreting tumor immune escape mechanisms. Typical “hot tumors” are characterized by abundant lymphocyte infiltration, high levels of inflammatory factor expression, and activation of antigen presentation mechanisms, forming an immune-supportive microenvironment (20). In contrast, “cold tumors” exhibit a lack of immune cell infiltration, accumulation of immunosuppressive cells, and the formation of physical barriers, leading to impaired immune surveillance (21). Although this classification concept is clearly defined at the theoretical level, a consensus on specific quantitative criteria is still lacking. This directly results in incomparable classification outcomes across different studies, thereby limiting the application of this classification method in research (22).

To establish a reproducible and standardized immune subtyping framework, this study utilized transcriptomic-derived lymphocyte infiltration quantification scores as the basis for classification. Through the ssGSEA approach (23), we systematically evaluated multiple immune cell subpopulations, including T cells, B cells, natural killer cells, and myeloid cells, to stratify LUAD patients into distinct high-immunity and low-immunity subgroups. This transcriptomic profiling-based strategy effectively captures key immunological features that differentiate “cold” from “hot” tumors (24) while providing a more objective, data-driven binary classification compared to traditional subjective approaches. Furthermore, it provides a more comprehensive representation of tumor immune microenvironment complexity, thereby enhancing the translational potential of our immune subtyping methodology for clinical applications.

By integrating computational pathology with transcriptomic profiling, we developed a novel immune classification model for LUAD that leverages quantitative analysis of TIL spatial distribution patterns within tumor stroma on H&E-stained WSIs. The model achieves accurate immune subtyping with high concordance to transcriptome-defined classifications, establishing a robust mapping between conventional histopathological features and transcriptomic profiles. This work provides an effective decision-support tool for guiding immunotherapy strategies, predicting treatment response, and facilitating personalized prognosis management in LUAD patients (25–27).

Recent studies have demonstrated the feasibility of predicting molecular characteristics from H&E-stained images using deep learning (28, 29). Fujii et al. (30) used this approach to predict BRAFV600E mutation and MSI-H status in colorectal cancer, two key markers for targeted therapy and immunotherapy. Leveraging TCGA data, multiple teams have trained models to predict various molecular phenotypes from histology. For instance, Yu et al. (31) applied a traditional pathomics workflow to HNSCC data, extracting 1,488 handcrafted features to predict IFNG expression levels. However, these studies primarily focus on individual gene mutations or expression levels. In contrast, our study targets a higher-level molecular phenotype, specifically immune subtypes defined by whole-transcriptome profiling. Rather than predicting isolated gene alterations, we establish a mapping between H&E patterns and transcriptome-defined immune classifications that capture the overall tumor immune microenvironment. This approach integrates histopathology with systems-level molecular phenotyping, offering a broader perspective for studying tumor immunology. Furthermore, by predicting a clinically relevant classification that distinguishes high- from low-immunity tumors, our model provides more direct guidance for immunotherapy decision-making.

Central to our approach is a modular design that employs deep learning exclusively for automated tissue annotation. Deep learning, and particularly convolutional neural networks (CNNs), has revolutionized computational pathology by enabling automated, high-throughput, and reproducible analysis of whole-slide images at scale (32, 33). A key advantage of CNNs lies in their ability to learn hierarchical representations directly from data. Lower layers capture basic features such as edges and textures, while deeper layers integrate these into complex morphological patterns corresponding to cells, glands, or tissue structures (34). This progressive learning capability enables CNNs to continuously refine their feature representations as they are exposed to more training data, leading to improved accuracy and robustness in tasks such as nuclei detection and tissue segmentation. Furthermore, when trained on diverse datasets encompassing variations in staining protocols, scanning resolutions, and tissue preparations, CNNs can learn to generalize across these technical heterogeneities. This capability is particularly advantageous when working with real-world multi-institutional data, such as the TCGA cohort used in this study (35). These characteristics make deep learning especially well-suited for tasks such as nuclei detection (35), tissue segmentation (36), and feature extraction, all of which are essential for quantitative pathology but challenging to perform manually at scale.

However, the manner in which deep learning is deployed determines whether its strengths are fully realized or its limitations become problematic. While deep learning excels at well-defined perception tasks, extending it to directly predict abstract clinical or molecular labels without intermediate interpretable steps results in end-to-end black-box models that, despite their technical feasibility, pose significant challenges for interpretability and limit clinical trustworthiness (37). This concern, however, pertains to the end-to-end paradigm rather than to deep learning technology itself, which can be harnessed as a powerful tool when deployed appropriately.

In contrast to approaches that apply deep learning directly to end-to-end prediction, our study employs it as a modular, interpretable tool for automated annotation (38, 39). We leverage deep learning models exclusively as automated annotation tools to generate visually interpretable outputs of histological structures, including tissue contours, tumor parenchyma, and lymphocytes. This strategy capitalizes on deep learning’s exceptional capability for pattern recognition and its ability to continuously learn and refine feature representations from large-scale annotated data (40), tasks where it consistently outperforms traditional rule-based image analysis methods, while keeping these modules fully transparent and verifiable. These models function as independently verifiable modules, producing annotations that can be directly inspected and validated by pathologists, rather than serving as a black-box predictor of immune subtypes. By utilizing the progressive learning capacity of convolutional neural networks for cellular recognition, this method significantly alleviates the extensive annotation burden common in TIME research. The generated fine-grained heatmaps additionally permit visual assessment of automated annotation accuracy, enhancing transparency at the annotation stage.

In the classification stage, we adopted a statistics-driven clustering methodology (41) rather than directly mapping image features to immune labels. Based on analysis of the training data distribution, we defined quantitative thresholds to evaluate spatial aggregation patterns of tumor-infiltrating lymphocytes and constructed classification rules according to sample distribution profiles. This modular design systematically separates the analytical workflow into independently verifiable stages (39), enabling pathologists to examine each procedural step from cellular annotation to spatial feature quantification. The framework thereby significantly improves both model transparency and clinical applicability. This hybrid approach allows us to benefit from both worlds (42), leveraging the high performance and scalability of deep learning for low-level perception tasks such as cell detection and tissue segmentation, while maintaining the transparency and interpretability of statistical rule-based systems for high-level decision-making in immune subtype classification. By confining deep learning to well-defined, observable tasks where outputs can be visually inspected and validated, we preserve the performance benefits of modern AI while ensuring that the final decision-making process remains transparent and clinically auditable.

The thresholds used in this study were initially derived from data-driven optimization in the training cohort. While such empirical threshold determination is common in computational pathology studies (43), it represents a methodological limitation, as threshold optimization ideally requires larger and more diverse datasets to ensure generalizability (44). Importantly, the validity of these thresholds is indirectly supported by the consistent and significant biological differences observed between the resulting high- and low-immunity groups across multiple independent validation dimensions, including genomic, transcriptomic, protein, spatial, and functional levels. The fact that the classification produced by these thresholds yields groups with distinct biological characteristics across diverse experimental modalities provides strong evidence that they capture genuine immunological heterogeneity, despite being derived from a limited training sample.

TMB has emerged as a clinically validated biomarker for immune checkpoint inhibitor response across multiple cancer types. Elevated TMB leads to increased neoantigen production, which can enhance tumor immunogenicity and drive more robust T cell infiltration and activation (45, 46). Clinical studies have demonstrated that patients with high TMB derive greater benefit from immunotherapy in cancers including lung adenocarcinoma (47), and TMB has consequently been incorporated into clinical guidelines as a predictive biomarker for immunotherapy decision-making (48). In this context, we examined the genomic characteristics of model-predicted immune subtypes. Our analysis revealed that cases classified as high-immunity by our model exhibited significantly higher TMB compared to low-immunity cases. This finding aligns with the established paradigm that increased neoantigen load drives immune recognition and lymphocyte infiltration.

The mechanistic link between TMB and TIL spatial patterns further strengthens this interpretation. High TMB tumors generate greater neoantigen diversity, which can trigger broader and more intense T cell responses, a mechanism that likely underlies the dense, aggregated TIL patterns captured by our high-attention regions. This genomic-immune coupling reinforces that our model-defined high-attention regions represent not merely lymphocyte aggregates, but immunologically active tumor microenvironments shaped by underlying mutational landscapes (49). Critically, the association between model-predicted immune subtypes and TMB provides genomic-level evidence that our morphology-based model captures clinically relevant immune phenotypes. Because high TMB is an established predictor of immunotherapy response, the significant TMB elevation in our model-predicted high-immunity group suggests that these patients may represent a population more likely to benefit from immune checkpoint inhibition (50). While direct validation in treatment cohorts remains necessary as a key limitation of the current study, this genomic correlation establishes a mechanistic bridge between our cost-effective H&E-based classification and clinically actionable biomarkers, supporting the potential utility of our model as a tool for immunotherapy decision-making, particularly in resource-limited settings. The genomic landscape underlying these spatial patterns provides an additional dimension of biological validation.

This study demonstrates that the spatial distribution patterns of lymphocytes can effectively predict the overall status of the TIME as defined by transcriptomic profiling. The biological foundation of this approach lies in the central role of TILs in tumor immunity and the systematic representation of immune status through their spatial organization.

From a functional perspective, TILs consist of multiple immune cell populations, including CD8^+^ and CD4^+^ T cells, regulatory T cells, B cells, and NK cells (51), which serve as the primary effectors of antitumor immune responses (52). Our analysis of immune cell infiltration revealed that CD4^+^ and CD8^+^ T cells not only constitute the predominant immune populations but also exhibit distinct morphological features in H&E-stained sections. Critically, their spatial distribution patterns most accurately reflect the functional state of the overall immune microenvironment. Molecular validation in our external cohort further supported this finding, as the model-predicted high-immunity group showed significant upregulation of both the cytotoxic marker CD8A (53) and the effector molecule GZMB (54), confirming enhanced functional activity of effector T cells at the molecular level.

In terms of regulatory mechanisms, TILs recruit additional immune components such as dendritic cells and macrophages through cytokine secretion including IFN-γ (55, 56). Moreover, they amplify antitumor immune responses by modulating cytokine networks and immune checkpoint molecules (57), thereby establishing localized immune activation units through these cascading effects. Protein-level evidence from the external validation cohort corroborates this regulatory pattern. The model-predicted high-immunity group demonstrated not only increased densities of CD3^+^ and CD8^+^ T cells but also concurrent infiltration of CD20^+^ B cells and CD68^+^ macrophages. This coordinated multicellular infiltration confirms that regions defined by lymphocyte distribution indeed form functional immune activation units, establishing a biological link between TILs spatial patterns and the comprehensive immune landscape.

Methodological selection was equally crucial for successful model development. The hierarchical clustering and dynamic threshold optimization strategy (41) employed in this study effectively captured the complex nonlinear relationships between lymphocyte spatial aggregation patterns and immune activation status. By quantifying the high-attention region ratio metric, we achieved precise characterization of tumor immune microenvironment heterogeneity, overcoming the limitations of conventional linear models in representing spatial complexity.

Collectively, these essential components including functional significance, regulatory mechanisms, and methodological innovation establish a robust biological foundation and create an effective technical framework for noninvasive assessment of the overall immune microenvironment status through spatial distribution analysis of TILs.

This study has several limitations. First, the WSIs from the TCGA database used for model training exhibit significant quality heterogeneity, including variations in staining protocols across institutions and inconsistencies in scanning resolutions. Although algorithmic preprocessing was applied to standardize image background and color, these inherent variations may still affect the accuracy of feature extraction and target recognition.

Second, while our IHC analysis confirmed quantitative differences in immune cell infiltration between model-predicted high- and low-immunity groups, it did not provide direct spatial evidence that cells within the high-attention regions are in a functionally active state. This represents a limitation of the current study, as functional activation is a key determinant of effective antitumor immunity. In future work, we plan to employ multiplex immunofluorescence techniques to spatially characterize the functional status of immune cells within these regions and to directly validate whether the high-attention regions identified by our model correspond to immunologically active microenvironments.

Third, this study primarily focuses on methodological development and validation, aiming to establish a low-cost and scalable tool that enables prediction of transcriptome-based immune subtypes using routine H&E-stained pathology sections. The observed association between model-predicted high-immunity subtypes and established immunotherapy-related biomarkers (e.g., high TMB, elevated CD8A expression) provides preliminary evidence for their potential relevance to immunotherapy response. However, the actual predictive value of the model for immunotherapeutic outcomes, such as objective response rate (ORR) or progression-free survival (PFS), remains to be validated in future prospective treatment cohorts. Future studies integrating clinical response data are necessary to translate these biological insights into actionable clinical decisions. These limitations collectively indicate promising directions for future investigation, including advanced algorithm development, integration of multimodal data sources, and expansion of sample cohorts. Our research team remains dedicated to further exploration in this field, with sustained focus on advancing computational pathology methodologies.

## 5 Conclusions

This study presents a modular computational pathology framework that integrates deep learning-based automated annotation with statistics-driven classification to predict transcriptome-defined immune subtypes from routine H&E-stained WSIs in LUAD. By confining deep learning to transparent annotation tasks and employing interpretable statistical rules for decision-making, our approach achieves both computational efficiency and clinical interpretability. This framework provides an efficient prediction tool for immunotherapy decision-making, offering a practical alternative in clinical scenarios where rapid or low-cost immune phenotyping is needed.

## Data Availability

The transcriptomic, genomic, and whole-slide image data from the TCGA-LUAD cohort are publicly available without restrictions under the TCGA Data Use Policy. The clinical and immunohistochemistry data from the external validation cohort are not publicly available due to patient privacy and ethical regulations but are available from the corresponding author upon reasonable request, subject to approval by the Ethics Committee of The Second Affiliated Hospital of Guangxi Medical University.
