# Supplementary material for: Development and validation of an interpretable prediction model using spatial patterns of tumor-infiltrating lymphocytes in H&E-stained whole-slide images for immune subtyping of lung adenocarcinoma
Source: Front Immunol. 2026 May 8;17:1773927. doi: 10.3389/fimmu.2026.1773927 (PMC13194154; doi:10.3389/fimmu.2026.1773927)
Supplement: Supplementary file 1 [file DataSheet1.doc]

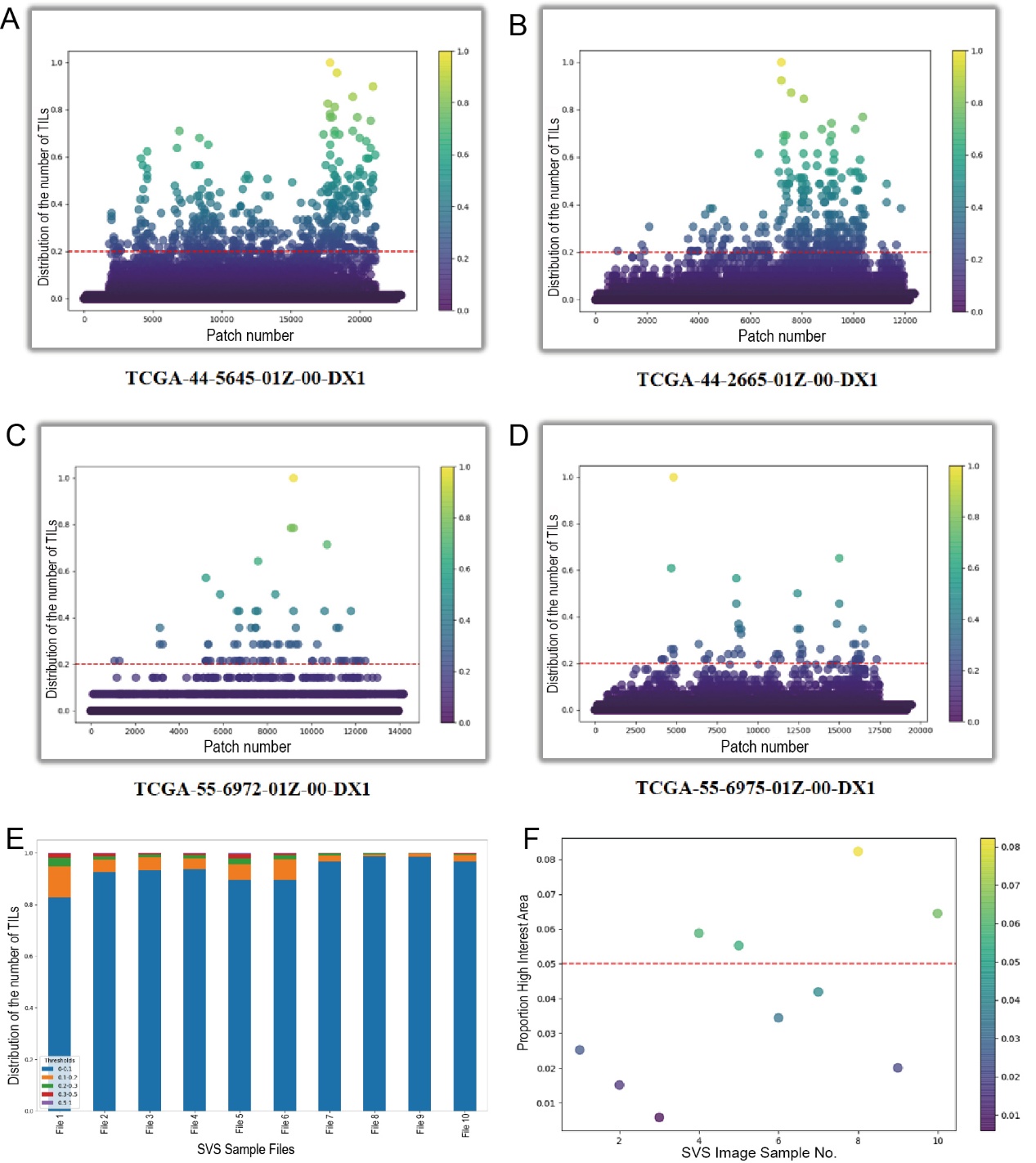


Figure S1. Determination of threshold values for immune subtype classification. (A-D) Normalization of the number of TILs in patch. Scatter plot of normalized TILs density. Each point represents the relative TIL density within a 100μm × 100μm patch of tissue from a WSI. (E) TILs number distribution percentage stacked histograms. Results indicate that intervals with a threshold greater than 0.2 exhibit a sparser and more stable data distribution, providing a basis for threshold selection. (F) Analysis of the percentage of TILs high-density image patchs. The optimal classification threshold was determined to be 0.05 through performance optimization (indicated by the dashed line).
